# The Use of Electrical Nerve Stimulation to Treat Migraines: A Systematic Review

**DOI:** 10.7759/cureus.17554

**Published:** 2021-08-30

**Authors:** Karan Patel, Sai Batchu, Rebecca Wang, Sean Bunachita, Aditya Joshi, Ria Soni, Aadi Pandya, Urvish Patel

**Affiliations:** 1 Medicine, Cooper Medical School of Rowan University, Camden, USA; 2 Medicine, Hackensack Meridian School of Medicine, Nutley, USA; 3 Medicine, Johns Hopkins University School of Medicine, Baltimore, USA; 4 Orthopaedics, Cooper Medical School of Rowan University, Camden, USA; 5 Medicine, Rutgers University, New Brunswick, USA; 6 Medicine, Herricks High School, New Hyde Park, USA; 7 Epidemiology and Public Health, Icahn School of Medicine at Mount Sinai, New York, USA

**Keywords:** migraine disorder, transcutaneous electrical nerve stimulation, vagus nerve stimulation, occipital nerve stimulation, percutaneous electrical nerve stimulation, trigeminal nerve stimulation

## Abstract

Migraines have been defined as an intense unilateral throbbing or pulsating sensation lasting anywhere between a few hours to multiple days. They are the sixth most prevalent disease in the United States, with approximately 18% of women and 6% of men experiencing some form of a migraine throughout their lifetime. In addition, they pose a significant economic burden, accounting for anywhere between $13 and $17 billion in medical costs annually in the United States. While there are a wide variety of treatments for migraines on the market, such as nonsteroidal anti-inflammatory drugs (NSAIDS), beta-blockers, and anti-epileptics, there is still no standard treatment. Moreover, each of these medications has a wide range of side effects, ranging from stomach ulcers to light-headedness. Within the last few decades, the presence of electrical nerve stimulation has emerged as a possible treatment option. These methods are almost free of harmful side effects and may be able to reduce the economic burden on those who suffer from migraines. However, studies have shown mixed results in regard to their efficacy. In this paper, we performed a systematic review to detail the current state of the literature regarding electrical nerve stimulation as a treatment modality for migraines.

## Introduction and background

Like most disorders, there appear to be both environmental and genetic factors that may account for the pathophysiology of migraines. A study conducted by Russel et al. showed that there was a higher rate of migraines among monozygotic twins than those experienced by dizygotic twins [1]. In addition, various mutations have been linked with different types of headaches. For example, a mutation in methylenetetrahydrofolate reductase, C667T, has been associated with aura positive migraines. Moreover, variations in chromosome 19p13, in the CACNA1A gene coding for a voltage-gated calcium channel, and mutations in the ATP1A2 gene have been found to be linked to familial hemiplegic migraines [1]. Environmental triggers of migraines include a wide variety of factors but are relatively shared among those who experience migraine attacks. These include, but are not limited to, bright lights, flickering lights, air quality changes, and odors [2].

Although the exact mechanism of migraines is not completely understood, there has been some progress made over the last few decades. Because vasoconstrictors had been shown to relieve migraine symptoms, it was previously postulated that migraines were a result of vasodilation [3]. However, more recent evidence has suggested that migraine pathophysiology may be far more nuanced than previously expected. The current understanding is that a pathway starting with afferent fibers from the occipital branch of the trigeminal nerve which eventually leads to third-order neurons from the basal ganglia, thalamus, hypothalamus synapsing onto cortical areas may be responsible for causing many of the migraine-related symptoms [4]. Moreover, even though migraines are common, various factors can also complicate their diagnosis. For example, cervicogenic headaches are often misdiagnosed as migraines. These headaches occur because of referred pain from the neck and share many features with migraine symptoms including unilateral head pain, photophobia, nausea, and vomiting [5]. Although we have made significant strides in our understanding of the pathophysiology and our ability to distinguish migraines from other similar headaches, more research still needs to be done to determine the precise mechanism of migraines and further distinguishing symptoms separating various types of headaches.

**Figure 1:**
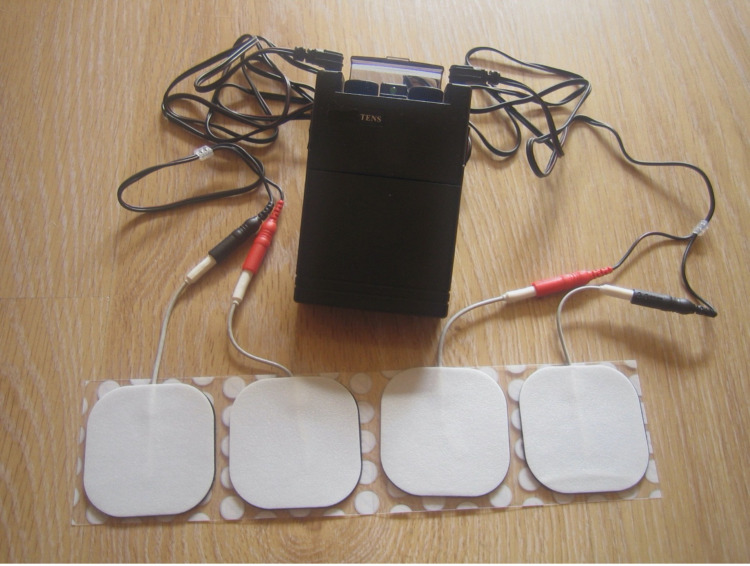
Transcutaneous electrical nerve stimulator Original image by Wikipedia user *Yeza*, distributed under a CC BY-SA 4.0 license. No modifications were made [9].

Despite the fact that current medications such as nonsteroidal anti-inflammatory drugs (NSAIDs) and topiramate have proved effective in treating migraine disorders, they also come with a high financial cost and a wide array of side effects [6, 7]. As a result, there continues to remain an investigation into other treatment modalities for migraines and their associated symptoms. One potential affordable therapeutic alternative may be electrical nerve stimulation (ENS). This treatment modality, which has various subclasses, delivers a voltage-driven electrical signal to a specific region of the body (which changes based on the type of ENS used). Essentially, this voltage-based impulse is thought to modulate the activity of various neurotransmitters including serotonin, gamma-aminobutyric acid (GABA), dopamine, and others. The electrical modulation of neurotransmitters changes the pattern of firing of neurons and can modulate pain, frequency, and other factors classically associated with migraines [8]. An example of a transcutaneous electrical nerve stimulator, one subcategory of electrical nerve stimulators, can be seen in Figure 1.

The studies on ENS have shown to have mixed results, with some studies demonstrating that they are effective, while others have shown that they were no more effective than sham treatments [10-12]. Yet recently, there have been some studies that have shown ENS to be effective from a neurological perspective. A study conducted by Zhang et al. found that there was a significant decrease in pain regions of the brain such as the locus coeruleus, in patients who received ENS as an adjunct treatment for their chronic migraines compared to those who received the sham treatment [13]. In addition, in a follow-up study, with an altered protocol, Zhang et al.’s fMRI study showed that transcutaneous auricular vagus nerve stimulation modulates the activity of thalamic circuits in patients suffering from migraines. Moreover, a recent study showed ENS can decrease pro-inflammatory cytokines, more specifically interleukin-1 β (IL-1β), which have been shown to be elevated in some types of headaches [11, 14]. These results prove encouraging in that they demonstrate both a cortical and chemical basis for the use of ENS in treating migraines.

In our study, we performed a systematic review of randomized controlled trials (RCTs) of ENS over the last five years. More specifically, we looked into studies that used various forms of ENS including occipital nerve stimulation (ONS), transcutaneous electrical nerve stimulation (TENS), vagus nerve stimulation (VNS) trigeminal nerve stimulation (TNS), and percutaneous electrical nerve stimulation (PENS). We wanted to determine if improvements in technologies combined with our increased knowledge on the pathophysiology of migraines over the last half decade have now allowed for ENS to become a viable alternative treatment for migraines.

## Review

Methods

We searched the MEDLINE database using PubMed. In addition, we searched the Embase, MEDLINE, and ClinicalTrials.gov databases using The Cochrane Library for RCTs with the search terms Electrical nerve stimulation OR ENS AND migraines, Occipital nerve stimulation or ONS AND migraines, Transcutaneous electrical nerve stimulation or TENS AND migraines, Vagus nerve stimulation AND migraines, Trigeminal nerve stimulation AND migraines, and Percutaneous electrical nerve stimulation OR PENS AND migraines. Outside of our manual search, we used SWIFT-Review to supplement the systematic review of clinical trials with ENS and migraines appearing in PubMed-indexed peer-reviewed literature [15]. Our reason behind only including studies from 2015 to 2021 was that we wanted to assess the current state of ENS in the treatment of migraines. Over the last three decades, the data on ENS as a treatment modality has been inconclusive with some studies showing that they have a positive impact, while other studies having found them to be no more beneficial than control sham treatments [10-12]. With the ever-evolving technologies, we aimed to assess if improvements in electrical nerve stimulators over the last five years have now allowed for them to serve as viable treatment options for migraines.

We included papers with the following criteria:

*Population*: Any person above the age of 18 who experiences migraines.

*Intervention*: Use of ENS.

*Comparison*: ENS versus sham or subthreshold stimulation. 

*Outcome*: ENS effect on number, frequency, or severity of migraines compared to sham treatments.

**Figure 2:**
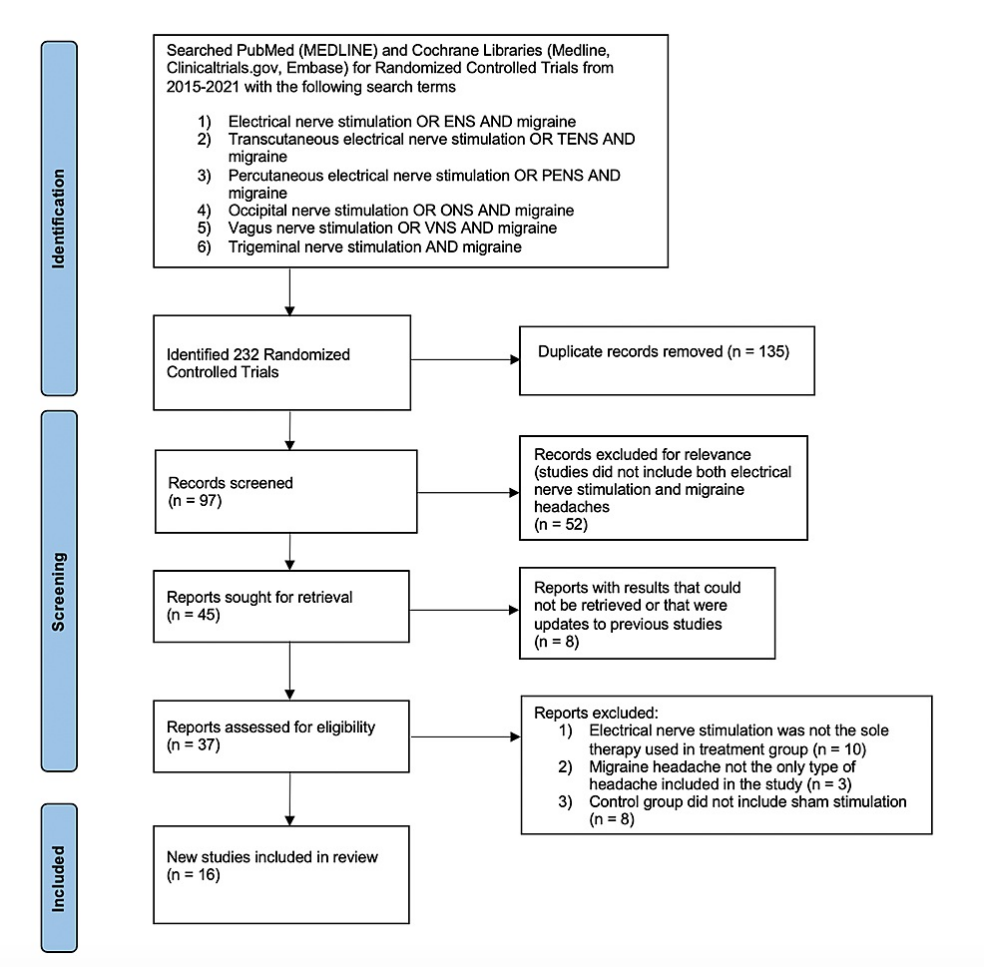
Search Strategy

A full list of our search strategy, with details on the articles that we included/excluded, can be seen below in Figure 2, which was generated using Prisma protocol [16]. In addition, Table 1 provides a comprehensive summary of each of the RCTs that we included in our study and their primary results. In order to assess each of the studies for potential bias, we used the Cochrane Collaboration's risk of bias tool and included our justification for any category we deemed to have anything other than low bias at the bottom of the Table 2 [17].

 

**Table 1:** Summary of included randomized control trials PENS: percutaneous electrical nerve stimulation; REN: remote electrical neuromodulation; TNS: trigeminal neurostimulation; TENS: transcutaneous electrical nerve stimulation; VAS: visual analog scale; ONS: occipital nerve stimulation; nVNS: non-invasive vagus nerve stimulation; tONS: transcutaneous occipital nerve stimulation; VNS: vagus nerve stimulation

Author/Year	Intervention/Control	Result
Li and Xu 2017 [18]	Percutaneous electrical nerve stimulation versus (PENS) sham treatment	Significant decrease in monthly migraine days, monthly headache attacks, and use of antimigraine drugs with electrodes were placed at the bilateral Taiyang points.
Yarnitsky et al. 2019 [19]	Remote electrical neuromodulation (REN) versus sham	REN, which was placed on the upper lateral portion of the arm, was more effective than sham in reducing pain associated with migraines and the “most bothersome symptoms” of migraines. Pain-free percentages were 37.4% REN vs. 18.4% sham, and most bothersome symptom relief percentages were 46.3% REN vs. 22.2% sham
Chou et al. 2019 [20]	Trigeminal neurostimulation (TNS) versus sham	Significant difference in pain reduction in those who suffered migraine without auras, but no difference in pain reduction for those who suffered from migraine with auras. Changes in the visual analog scale (VAS) for migraine without auras were -3.3 ± 2.4 for the TNS group versus -1.7 ± 1.9 for the sham group
Hokenek et al. 2021 [21]	TENS versus sham	Significant decrease in pain intensity associated with migraines in the TENS group compared to the sham group. Changes in VAS scores were as follows: -65 ± 25 for TENS and -9 ± 2 for the sham group
Mekhail et al. 2017 [22]	ONS versus sham	Average reduction of 8.51 headache days per month in ONS group compared to sham
Dodick et al. 2015 [23]	ONS versus sham	Average reduction of 6.7 +/- 8.4 headache days per month; 65.4% of participants reported excellent or good headache relief with ONS compared to sham
Slotty et al. 2015 [24]	ONS versus subthreshold stimulation	Significant improvements reported in pain threshold of suprathreshold stimulation versus subthreshold (1.98 ± 1.56 vs. 5.65 ± 2.11) on the VAS
Danno et al. 2019 [25]	Supraorbital transcutaneous nerve stimulation versus sham	Significant decrease in the number of migraine attacks (5.33 vs. 3.94), in the number of migraine days per month (8.16 vs. 6.84), decrease in number of days per month a patient takes migraine drugs (8.75 vs. 7.83) in the supraorbital transcutaneous stimulation group
Tassorelli et al. 2018 [26]	Non-invasive vagus nerve stimulation (nVNS) versus sham	nVNS was superior to sham in reducing pain at 30 minutes (12.7% vs 4.2%; p = 0.012) and 60 minutes (21.0% vs 10.0%; p = 0.023), but not at 120 minutes (30.4% vs 19.7%; p = 0.067). Total pain-free percentage difference between the nVNS group and sham group was 13.7%
Diener et al. 2019 [27]	nVNS versus sham	Mean reduction in headache days per month was 2.27 for the nVNS group compared to 1.53 for the sham group (p = 0.0043)
Blech et al. 2020 [28]	nVNS versus sham	No significant differences were found at 120 minutes in pain freedom between sham and nVNS group (30.4% for nVNS vs. 19.7% for sham; p = 0.067). Significant differences were found in pain freedom between the two groups at 30 minutes and 60 minutes and greater pain relief was reported in the nNVS group at 120 minutes
Grazzi et al. 2018 [29]	nVNS versus sham	Significant decrease in pain intensity at 30 minutes (nVNS, 32.2%; sham, 18.5%; p = 0.020), 60 minutes (nVNS, 38.8%; sham, 24.0%; p = 0.017), and 120 minutes (nVNS, 46.8%; sham, 26.2%; p = 0.002). Decrease in those who required medication for their migraines (nVNS, 59.3%; sham, 41.9%; p = 0.013)
Martelletti et al. 2018 [30]	nVNS versus sham	Patients in the nVNS group reported more pain-free attacks with 60 minutes (p = 0.025) and 120 minutes of treatment (p = 0.018) compared to the sham group. There was also significant reduction in pain associated with migraines for both those in the 60- and 120-minute treatment groups (p = 0.029 vs. 0.011, respectively) compared to the sham group
Silberstein et al. 2016 [31]	nVNS versus sham	Mean change in the nNVS group in headache days was -7.9 (95% confidence interval: -11.9 to -3.8; p < 0.01)
Liu et al. 2017 [32]	Transcutaneous occipital nerve stimulation (tONS) (2 Hz, 2 Hz/100 Hz,100 Hz) versus sham versus topiramate	Endpoints were reported as follows: headache days (significant reductions in both the 100 Hz tONS group and topiramate), duration, intensity (equal among all five groups: 2 Hz, 2 Hz/100 Hz,100 Hz, sham, and topiramate), and 50% responder rates (significant reductions equal among all tONS stimulation groups and topiramate)
Lendvai et al. 2020 [11]	VNS versus sham	Decreased number of severe migraine attacks per month, but no decreases in total attacks/month or headache days/month between VNS and sham groups

**Table 2:** Cochrane Collaboration's risk of bias Justification for any categories not receiving “low risk of bias” * Had an open-label phase of the study ** 70% of participants reported having an adverse event, with 40.7% requiring hospitalization

Author name/year	Random sequence generation (selection bias)	Allocation concealment (selection bias)	Blinding of participants and personal (performance bias)	Blinding of outcome Assessments	Incomplete data outcome	Selective reporting	Other bias
Li and Xu 2017 [18]	Low risk of bias	Low risk of bias	Low risk of bias	Low risk of bias	Low risk of bias	Low risk of bias	Low risk of bias
Yarnitsky et al. 2019 [19]	Low risk of bias	Low risk of bias	Low risk of bias	Low risk of bias	Low risk of bias	Low risk of bias	Low risk of bias
Chou et al. 2019 [20]	Low risk of bias	Low risk of bias	Low risk of bias	Low risk of bias	Low risk of bias	Low risk of bias	Low risk of bias
Hokenek et al. 2021 [21]	Low risk of bias	Low risk of bias	Low risk of bias	Low risk of bias	Low risk of bias	Low risk of bias	Low risk of bias
Mekhail et al. 2017 [22]	Low risk of bias	Low risk of bias	Low risk of bias	Low risk of bias	Low risk of bias	Low risk of bias	Unknown risk of bias*
Dodick et al. 2015 [23]	Low risk of bias	Low risk of bias	Low risk of bias	Low risk of bias	Low risk of bias	Low risk of bias	Unknown risk of bias**
Slotty et al. 2015 [24]	Low risk of bias	Low risk of bias	Low risk of bias	Low risk of bias	Low risk of bias	Low risk of bias	Low risk of bias
Danno et al. 2019 [25]	Low risk of bias	Low risk of bias	Low risk of bias	Low risk of bias	Low risk of bias	Low risk of bias	Low risk of bias
Tassorelli et al. 2018 [26]	Low risk of bias	Low risk of bias	Low risk of bias	Low risk of bias	Low risk of bias	Low risk of bias	Low risk of bias
Diener et al. 2019 [27]	Low risk of bias	Low risk of bias	Low risk of bias	Low risk of bias	Low risk of bias	Low risk of bias	Unknown risk of bias
Blech et al. 2020 [28]	Low risk of bias	Low risk of bias	Low risk of bias	Low risk of bias	Low risk of bias	Low risk of bias	Low risk of bias
Grazzi et al. 2018 [29]	Low risk of bias	Low risk of bias	Low risk of bias	Low risk of bias	Low risk of bias	Low risk of bias	Low risk of bias
Martelletti et al. 2018 [30]	Low risk of bias	Low risk of bias	Low risk of bias	Low risk of bias	Low risk of bias	Low risk of bias	Unknown risk of bias*
Silberstein et al. 2016 [31]	Low risk of bias	Low risk of bias	Low risk of bias	Low risk of bias	Low risk of bias	Low risk of bias	Unknown risk of bias*
Liu et al. 2017 [32]	Low risk of bias	Low risk of bias	Low risk of bias	Low risk of bias	Low risk of bias	Low risk of bias	Low risk of bias
Lendvai et al. 2020 [11]	Low risk of bias	Low risk of bias	Low risk of bias	Low risk of bias	Low risk of bias	Low risk of bias	Low risk of bias

We attempted to conduct a meta-analysis on the studies included in our systematic review, however this was not possible because of the heterogeneity of the studies, as we focused on all types of ENS rather than one in particular. However, in the future we plan on conducting a more detailed follow-up analysis of each type of electrical nerve stimulation individually. We will also not limit our search to RCTs, and instead include all types of studies.

Results

Discussion

Based on all of the studies included in our systematic review, there was a near-consensus that ENS had a significant therapeutic benefit for those who suffer from chronic migraines. Numerous studies in our analysis reported decreases in the VAS, mean number of headache days, severity of attacks, and pain associated with migraines in the groups that received ENS treatment compared to those who received sham stimulation [18-32]. Only two studies reported no significant differences between ENS and sham in one or more of their endpoints [11, 20]. Interestingly, one of these two studies found no significant differences only in the subgroup of patients who suffered migraines with auras. In the other subgroup, those who suffered from migraines without auras, ENS was shown to have a therapeutic effect [20]. The other studies included in our systematic review did not draw a distinction between the subtypes of migraines. As a result, it is still yet to be determined if these results were due to natural variation in the population analyzed or a result of the differences in pathophysiologies of migraines with and without auras.

Of note, a study conducted by Liu et al. found that using a 100 Hz frequency to treat migraine-associated symptoms had similar results to using topiramate [32]. Additionally, a study conducted by Rapoport et al. also presented similar findings. The researchers in this study determined a non-inferiority of REN compared to the conventional pharmacological treatments in completely relieving all migraine-associated pain [33]. While more studies of this nature are needed, specifically non-inferiority trials, to corroborate these findings, the results of these studies are rather encouraging. Among the most prevalent issues in migraine disorders are drug-induced side effects. Chronic use of drugs such as aspirin can cause stroke, gastrointestinal bleeding, and stomach ulcers while chronic topiramate use can cause blurry vision, fatigue, and memory issues [6, 7]. ENS may serve a beneficial role in that it not only has a very narrow side effect profile outside of hardware malfunctions, but also has very few contraindications [34]. In addition, ENS units may help to drive down costs associated with chronic migraine symptoms. After all, on average, a TENS unit ranges from $20 to $100 in price and is a one-time-only purchase [35] Furthermore, another study found that an ONS unit reduces cost by $1,577 annually compared to conventional treatments for certain types of headaches [36]. In contrast, the current average cost of chronic migraine treatment ranges between $8,500 and $9,500 and accounts for $13 to $17 billion in the United States annually [37, 38].

Overall, the positive results found in our study may stem from improvements in technologies and our understanding of both the mechanisms behind ENS and the pathophysiology of migraines. As mentioned above, ENS may be administered through a wide range of frequencies (from 1 Hz to 100 Hz). These wide ranges of frequencies work through various different mechanisms which may influence therapeutic outcomes. For example, in TENS, high-frequency stimulation is thought to inhibit pain according to the gate control theory of pain, while low-frequency stimulation is thought to work through the modulation of opioid receptors, GABA receptors, and serotonin receptors in the periaqueductal gray, rostral ventral medulla, and spinal cord [39, 40]. Previous studies that found inconclusive results may have used frequencies that were not therapeutically optimal. Only recently have we begun discovering which stimulation frequencies may be ideal to treat migraines. For example, in a direct comparison study published in 2015 between 1 Hz and 25 Hz frequencies, researchers found that using a 1 Hz frequency to treat migraines resulted in a significantly larger reduction in mean headache days compared to treatment with a 25 Hz frequency (-7.0 ± 4.6 vs. -3.3 ± 5.4 days) [41]​​. Furthermore, even though various methods of ENS, such as VNS, have been present for a while, the vast majority of RCTs regarding ENS have taken place during the last half decade. The data gathered as a result of these trials has allowed for a better understanding of the underlying mechanisms of ENS and their optimal settings.

## Conclusions

While more research needs to be conducted in order to fully understand the nuances of electrical nerve stimulators, they may indeed serve as a viable therapeutic intervention for migraines. Although previous studies have shown mixed efficacy with the use of ENS, technological advances and improvements in our clinical knowledge have resulted in significant improvements to electrical nerve stimulators. The results of our study, which included 18 separate RCTs, are encouraging in that it showed a near-consensus that ENS was effective. As a result, if this trend among studies continues, those who suffer from migraines will have an affordable treatment option, free of the negative side effects associated with commonly used migraine medications.
